# Clinical outcomes of two types of cages used in transforaminal lumbar interbody fusion for the treatment of degenerative lumbar diseases: n-HA/PA66 cages versus PEEK cages

**DOI:** 10.1007/s10856-016-5712-7

**Published:** 2016-04-18

**Authors:** Qian-xing Deng, Yun-sheng Ou, Yong Zhu, Zeng-hui Zhao, Bo Liu, Qiu Huang, Xing Du, Dian-ming Jiang

**Affiliations:** Department of Orthopedics, The First Affiliated Hospital of Chongqing Medical University, YouYi Road 1#, YuZhong District, Chongqing, 400016 People’s Republic of China

## Abstract

This study reports the clinical effects of nano-hydroxyapatite/polyamide66 cages (n-HA/PA66 cages) and compares the clinical outcomes between n-HA/PA66 and polyetheretherketone cages (PEEK cages) for application in transforaminal lumbar interbody fusion (TLIF). A retrospective and case–control study involving 124 patients using n-HA/PA66 cages and 142 patients using PEEK cages was conducted. All patients underwent TLIF and had an average of 2-years of follow-up. The Oswestry Disability Index and Visual Analog Scale were selected to assess the pain of low back and leg, as well as neurological status. The intervertebral space height and segmental angle were also measured to estimate the radiological changes. At the 1-year and final follow-ups, the fusion and subsidence rates were evaluated. There was no significant difference between the two groups regarding clinical and radiological results. At the final follow-up, the bony fusion rate was 92.45 and 91.57 % for the n-HA/PA66 and PEEK groups, respectively, and the subsidence rate was 7.55 and 8.99 %, respectively. The study indicated that both n-HA/PA66 and PEEK cages could promote effective clinical and radiographic outcomes when used to treat degenerative lumbar diseases. The high fusion and low subsidence rates revealed that n-HA/PA66 cages could be an alternative ideal choice as the same to PEEK cages for lumbar reconstruction after TLIF.

## Introduction

TLIF, which was described by Harms and Rolinger [1], is widely applied for the treatment of degenerative lumbar diseases with favorable outcomes [2–8]. One of the biggest advantages of TLIF is that it decreases the postoperative neurological deficit by reducing excessive neural tissue and dural sac retraction compared with posterior lumbar interbody fusion [9, 10]. Other advantages include avoiding potential complications associated with anterior lumbar interbody fusion, shorter hospital stays and lower costs compared with anterior combined with posterior approaches [11]. In addition, TLIF theoretically offers a lower risk of segmental instability because of the preservation of posterior lamina arch and posterior longitudinal ligament complex [9]. Typical indications for TLIF are degenerative or isthmus spondylolisthesis, degenerative disc disease, lumbar stenosis, lumbar disc herniation and recurrent lumbar disc herniation [9, 12].

Alternative materials for interbody fusion include auto-graft iliac crest, allograft bone, carbon fiber cages, titanium mesh cages, PEEK and n-HA/PA66 cages [4, 13–16]. Auto-graft iliac crest has been considered the ‘‘gold standard’’ for anterior column reconstruction, but there are some donor-site complications [4]. PEEK cages are radiolucent and have an elastic modulus similar to native bone [17]. PEEK cages augmented by pedicle screws have been shown to promote lumbar interbody fusion and to provide excellent clinical outcomes [3, 18]. The n-HA/PA66 cages are hollow bullets consisting of n-HA/PA66 composite, which simulates the constituent form of native bone [19–24]. In recent years, n-HA/PA66 cages filled with auto-graft bone have been reported to treat cervical spondylosis, cervical spondylotic myelopathy and thoraco-lumbar fractures with satisfactory clinical outcomes [15, 16, 25, 26]. However, to the best of our knowledge, there is a lack of literatures reporting the clinical application of n-HA/PA66 cages for the treatment of degenerative lumbar diseases, and there are little articles comparing the clinical efficacy of n-HA/PA66 cages and PEEK cages. Furthermore, the reconstruction and bony fusion of lumbar spine after discectomy remains challenge. The present retrospective study aimed to compare the clinical outcomes of n-HA/PA66 and PEEK cages used in TLIF. Thus, we make a hypothesis that n-HA/PA66 cages can lead to favorable clinical efficacy, and that both clinical and iconographic outcomes of n-HA/PA66 cages are corresponding to that of PEEK cages.

## Materials and methods

### Patients

The primary study subjects were patients who were diagnosed with degenerative or isthmus spondylolisthesis, degenerative disc disease, lumbar stenosis, lumbar disc herniation or recurrent lumbar disc herniation between August 2010 and December 2013. We excluded patients with lumbar tuberculosis, tumor or infection or trauma or who lacked sufficient clinical data. The patients were retrospectively divided into two groups based on cage types.

### Interbody cages

**Fig. 1:**
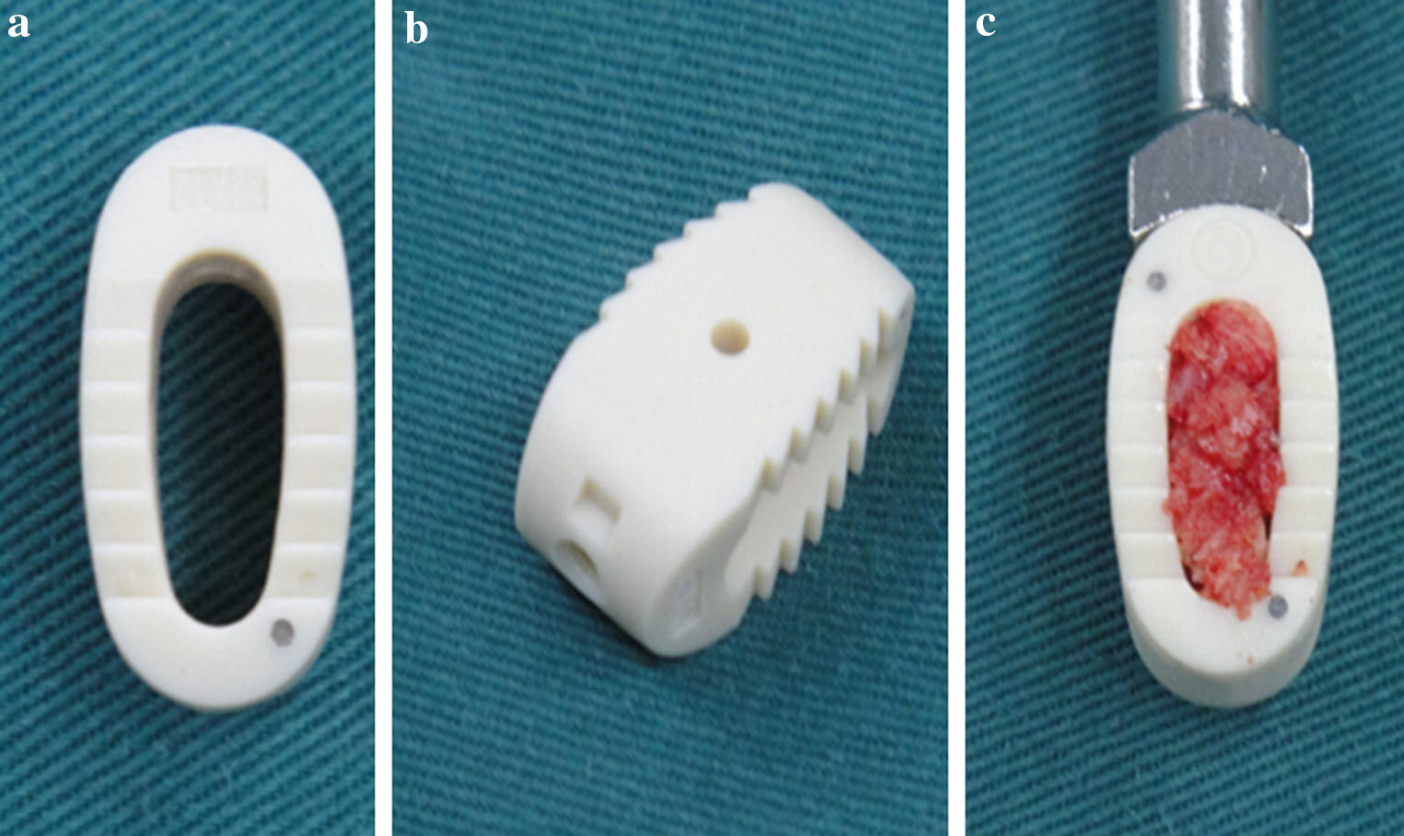
Photos of n-HA/PA66 cages: superior (**a**) and lateral (**b**) views and packed with osseous granula (**c**)

**Fig. 2:**
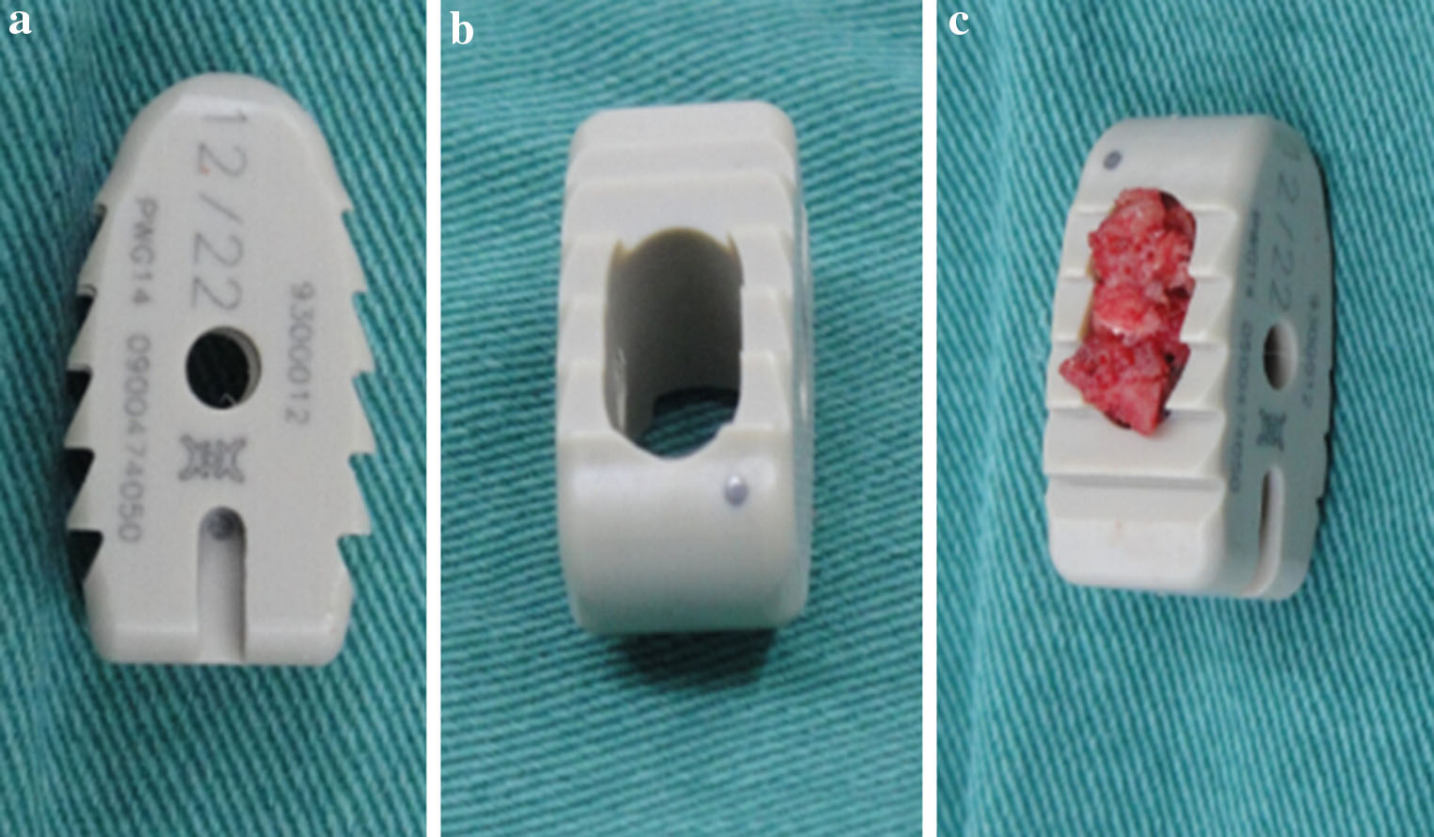
Photos of PEEK cages: superior (**a**) and lateral (**b**) views and packed with osseous granula (**c**)

The n-HA/PA66 cages were designed and fabricated by the Institution of Materials Science and Technology, Sichuan University, and our department (Fig. 1). The PEEK cages were from the Shandong We-go Orthopedic Group Medical Polymer CO., Ltd. Shandong, China (Fig. 2).

### Surgical procedures

All patients underwent preoperative examinations, including static and lateral flexion/extension radiographs and computed tomography scan. Neurogenic claudication, low back pain, and radicular symptoms were investigated with magnetic resonance imaging. TLIF was conducted as descriped by Meyer et al. [9]. In this protocol, we assumed that hypertrophic osteophytes surrounding the lateral recess and the ligamentum flavum were removed in every case to ensure that the dura mater was widely exposed and that the nerve root was released. The adjacent cartilage endplates were removed as fully as possible, but the bony endplates were preserved.

All patients were instructed to wear a lumbar brace for a period of approximately 12 weeks and had 3-, 6- and 12-month follow-ups, as well as a final follow-up. Static and lateral flexion/extension radiographs were used to assess the instruments, stability, lumbar curvature and disc-space height of the fused segments and bony fusion status. If necessary, CT scan was taken for further evaluation.

### Outcome measurements

**Fig. 3:**
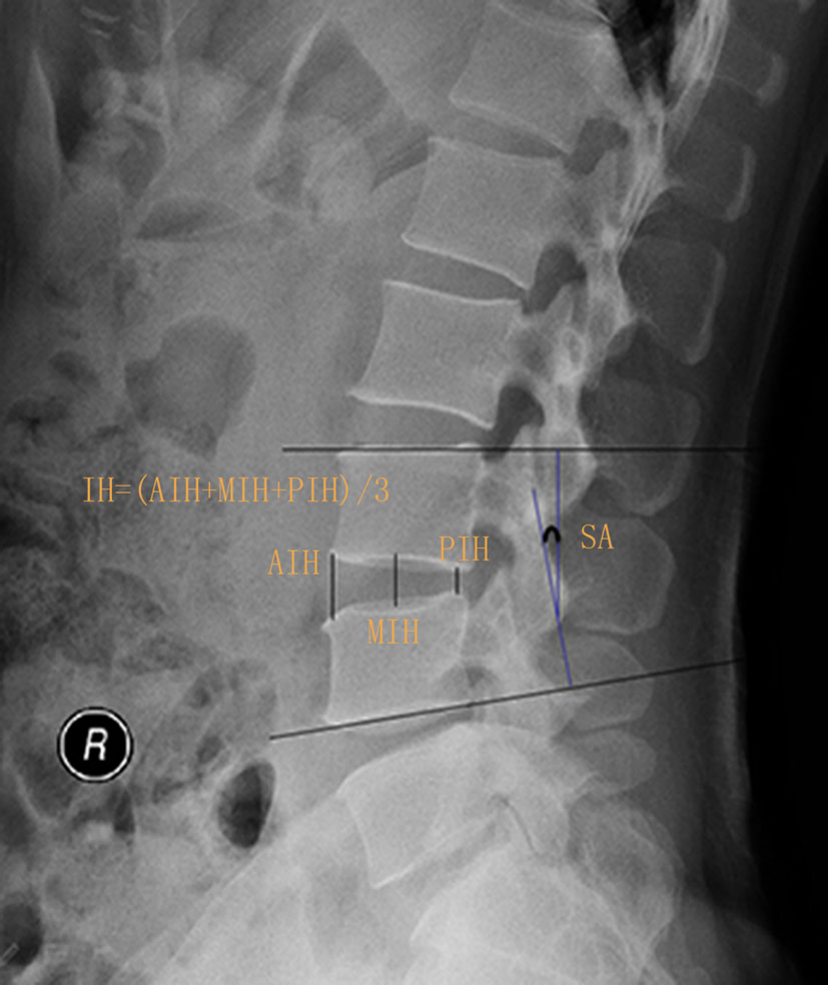
Methods to measure intervertebral space height and segmental angle. IH = (AIH + MIH + PIH)/3. *AIH* anterior intervertebral space height; *MIH* middle intervertebral space height; *PIH* posterior intervertebral space height. SA, between the superior endplate of upper vertebral and inferior endplate of lower vertebral of fused segment on neutral lateral lumbar plain film

The surgery time, blood loss, and perioperative complications were recorded. The ODI and VAS were applied to evaluate the pain of low back and leg, as well as neurological status at preoperative, 12-month and final follow-up time points. At the preoperative, 1-week, 3-, 6-, and 12-month and final follow-up time points, we measured the intervertebral space height (IH, Fig. 3) and segmental angle (SA, Fig. 3) using Carestream software (versions 10.0, Carestream Health, Eastman Kodak, Inc. Rochester, NY, USA). The loss of IH and SA was defined as the D-value between final and 1-week postoperative follow-ups. The cage subsidence was defined as any loss of IH more than 3 mm [27].

Bony fusion was identified by the following: the presence of trabeculation and bone bridging between cages and adjacent endplates, the absence of greater than 3 mm translational motion and more than 5° angular motion upon flexion/extension radiographs in the fused segments and the absence of a radiolucent gap between the cages and endplates [28]. If the surgeons were uncertain, three-dimensional computed tomography scans were taken to verify the fusion status by observing the trabeculation between the autogenous cancellous graft and adjacent endplates.

### Statistical analyses

All statistical analysis was performed using the Statistical Package for the Social Sciences version 17.0 (SPSS, Chicago, IL, USA). Quantitative data are presented as the mean ± standard deviation. Repeated measures ANOVA was used for statistical analyses of differences in mean values, and the Chi-squared test was used for categorical data between groups. The independent *t* test was applied to compare the clinical and radiological data of two cages. Significant difference was accepted at *P* < 0.05.

## Results

### Patient demographics

**Fig. 4:**
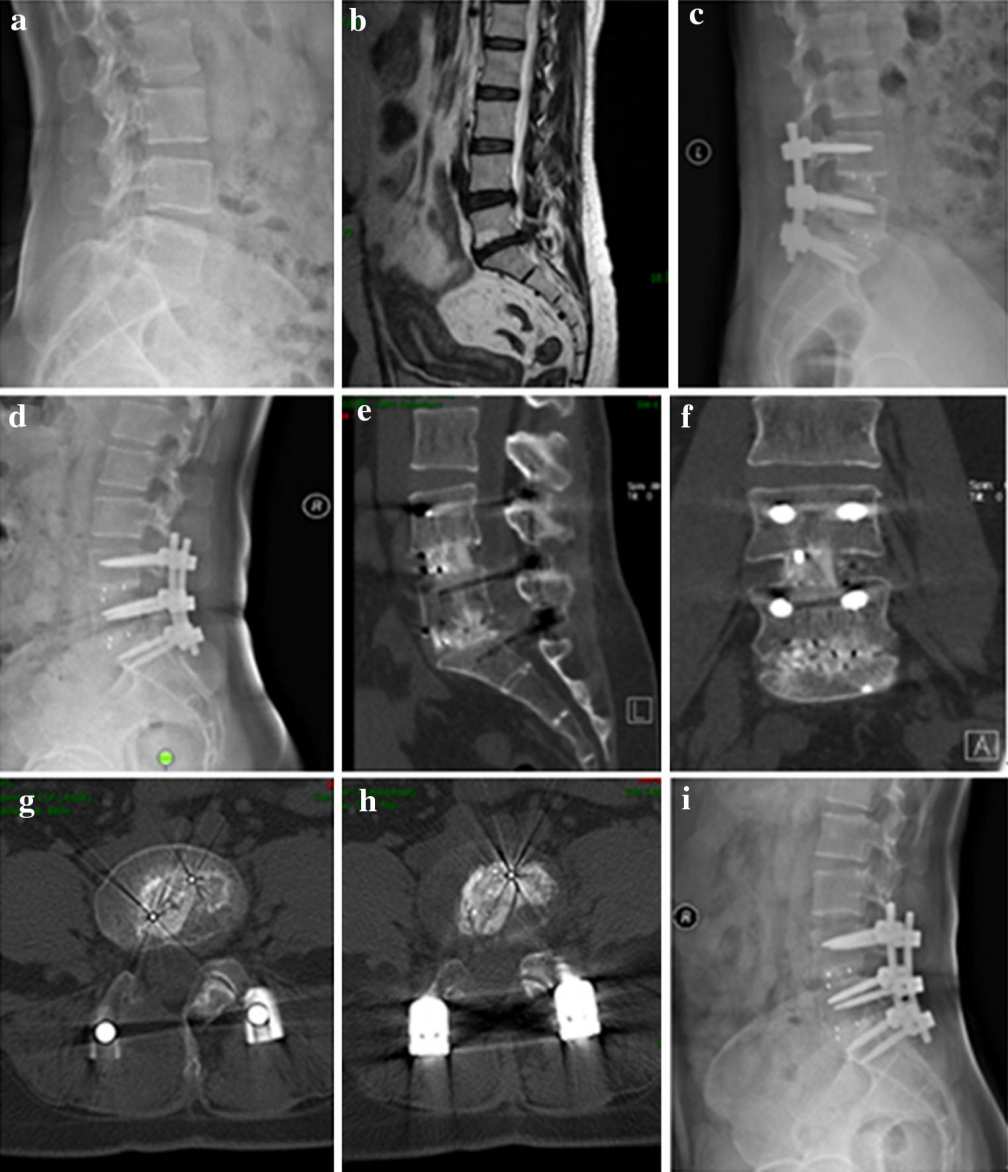
A 42-year-old female who underwent 2-level TLIF with n-HA/PA66 cages for lumbar reconstruction. The preoperative lumbar radiographs (**a**, **b**). The 1-week postoperative and 3-month follow-up radiographs (**c**, **d**). The CT or 3D-CT scan (**e**, **f**, **g**, **h**) shows that the autogenous bone granules fill the cages and achieve bony fusion with adjacent endplates by the 10-month follow-up. A lateral radiograph (**i**) at the final follow-up shows satisfactory bony fusion and no obvious migration, radiolucent gap or subsidence

**Fig. 5:**
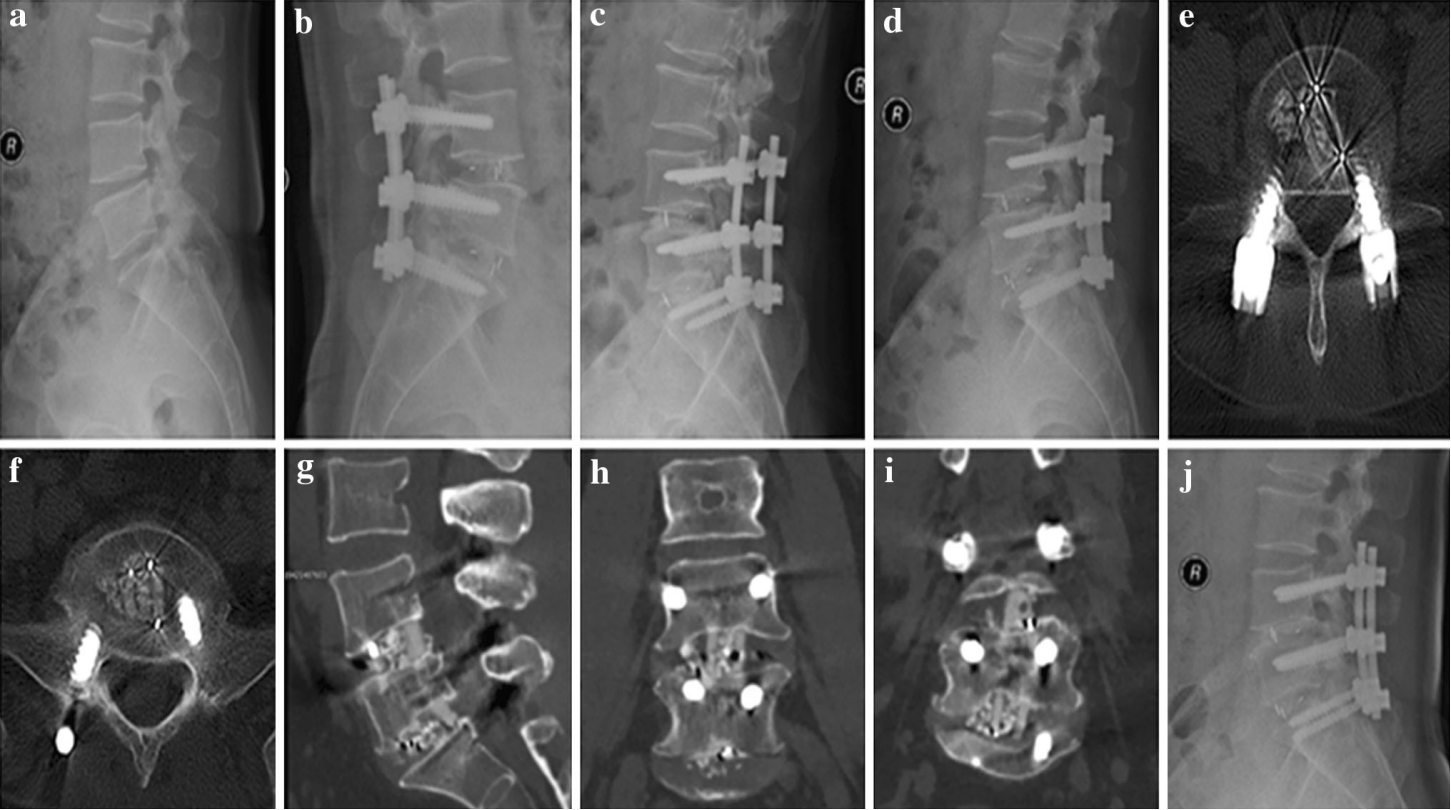
A 58-year-old male who underwent 2-level TLIF with PEEK cages for lumbar reconstruction. The preoperative radiograph (**a**). The 1-week postoperative and 3-month and 6-month follow-up radiographs (**b**, **c**, **d**). The CT or 3D-CT scan (**e**, **f**, **g**, **h**, **i**) shows that the autogenous bone granules fill the cages and achieve bony fusion with adjacent endplates by the 12-month follow-up. A lateral radiograph (**j**) at the final follow-up shows satisfactory bony fusion and no obvious migration, radiolucent gap or subsidence

A total of 266 patients with an average 24.24 ± 8.97 months of follow-up (range 12–47 months) were included in this study. Of these, 124 patients underwent TLIF with an n-HA/PA66 cage (Fig. 4) and 142 patients underwent TLIF with a PEEK cage (Fig. 5). The demographics of the patients were shown in Table 1. No significant differences were detected in gender, age, course of disease, surgery time, blood loss, or perioperative complications between the n-HA/PA66 and PEEK cage groups.

**Table 1:** The demographic and clinical data of patients

Parameters	n-HA/PA66 Cage (n = 124)	PEEK Cage (n = 142)	*P*
Male/female	61/63	60/82	0.257
Age	53.28 ± 12.51	53.65 ± 14.43	0.823
Course of disease	43.74 ± 60.95	44.68 ± 61.50	0.901
Diagnosis			
LDH	79	90	
LS	34	35	
LSS	6	6	
Revision	4	10	
LDS	1	1	
Surgery time	169.31 ± 34.25	164.82 ± 40.22	0.332
Blood loss	268.79 ± 193.52	236.69 ± 201.63	0.188
Perioperative complication	17/124 (13.71 %)	18/142 (12.68 %)	0.804
Follow up	23.94 ± 9.17	24.49 ± 8.81	0.619
Segments			
1-L,2-L,3-L	89/35/0	108/32/2	
L1/2,L2/3,L3/4,L4/5,L5/S1	0/2/16/86/55	4/1/21/89/63	

*LDH* lumbar disc herniation, *LS* lumbar spondylolisthesis, *LSS* lumbar spinal stenosis, *LDS* lumbar degenerative scoliosis

### Radiological outcomes

The IH was improved in the n-HA/PA66 group from 9.44 ± 2.16 mm preoperative to 12.62 ± 1.58 mm at 1-week postoperative and in the PEEK group from 9.28 ± 2.14 mm preoperative to 12.51 ± 1.72 mm at 1-week postoperative. The average correction of the IH was 3.18 ± 1.73 mm in the n-HA/PA66 group, and the mean loss of the IH was 1.65 ± 0.87 mm. There were no significant differences between the two groups for all of the above parameters at any time point observed (*P* > 0.05, Table 2). Regarding the SA, it also did not differ significantly (*P* > 0.05) except in the loss of SA (*P* = 0.044, Table 2).

**Table 2:** SA, IH, and fusion and subsidence rates at various time points (Mean ± SD)

Parameters	n-HA/PA66 Cage (n = 159)	PEEK Cage (n = 178)	*P*
SA (°)			
Pre-O	17.94 ± 8.49	16.91 ± 8.53	0.267
1-w Post-O	18.50 ± 6.09	18.54 ± 6.71	0.954
3-m Post-O	18.24 ± 6.00	18.10 ± 6.62	0.839
6-m Post-O	17.65 ± 5.64	17.47 ± 6.42	0.775
1-y Post-O	17.24 ± 5.66	16.91 ± 6.27	0.605
Fin-foll-up	16.82 ± 5.61	16.38 ± 6.11	0.495
Correction	0.55 ± 5.89	1.63 ± 4.91	0.072
Loss	1.68 ± 2.07	2.16 ± 2.26	0.044
IH (mm)			
Pre-O	9.44 ± 2.16	9.28 ± 2.14	0.490
1-w Post-O	12.62 ± 1.58	12.51 ± 1.72	0.536
3-m Post-O	11.95 ± 1.48	11.70 ± 1.47	0.114
6-m Post-O	11.55 ± 1.41	11.32 ± 1.36	0.141
1-y Post-O	11.27 ± 1.32	11.07 ± 1.39	0.177
Fin-foll-up	10.97 ± 1.26	10.86 ± 1.37	0.453
Correction	3.18 ± 1.73	3.23 ± 1.72	0.791
Loss	1.65 ± 0.87	1.65 ± 0.97	0.966
Fusion rate (%)			
1-y Post-O	139/159 (87.42 %)	155/178 (87.08 %)	0.925
Fin-foll-up	147/159 (92.45 %)	163/178 (91.57 %)	0.766
Subsidence rate (%)			
3-m Post-O	6/159 (3.77 %)	4/178 (2.25 %)	0.526
6-m Post-O	9/159 (5.66 %)	6/178 (3.37 %)	0.309
1-y Post-O	11/159 (6.92 %)	10/178 (5.62 %)	0.622
Fin-foll-up	12/159 (7.55 %)	16/178 (8.99 %)	0.632

*Pre*-*O* pre-operation, *Post*-*O* post-operation, *Fin*-*foll*-*up* final follow-up

At the 1-year follow-up, 87.42 % of patients in the n-HA/PA66 group and 87.08 % of patients in the PEEK group showed bony fusion. At the final follow-up, the bony fusion rate was 92.45 and 91.57 % in the n-HA/PA66 and PEEK groups, respectively. Three months after operation, the cage subsidence was 3.77 % in the n-HA/PA66 group and 2.25 % in the PEEK group. At 6 months as well as at 1 year and the final follow-up, there were no significant differences in the bony fusion and cage subsidence rates (*P* > 0.05, Table 2).

### Clinical outcomes

The preoperative ODI and VAS scores did not differ between the n-HA/PA66 and PEEK groups. Upon follow up, VAS scores had improved significantly for both groups, but no significant differences were found between the n-HA/PA66 and PEEK groups (*P* > 0.05, Table 3). The ODI was also similar between the two types of cage groups during follow up (*P* > 0.05, Table 3).

**Table 3:** VAS and ODI at pre-o and post-o (mean ± SD)

Parameters	n-HA/PA66 Cage	PEEK Cage	*P*
VAS (points)			
Pre-O	6.02 ± 1.20	6.17 ± 1.38	0.338
1-y Post-O	2.31 ± 0.85	2.25 ± 0.87	0.617
Fin-foll-up	1.56 ± 0.87	1.58 ± 0.89	0.853
ODI (%)			
Pre-O	50.56 ± 6.41	51.00 ± 6.47	0.583
1-y Post-O	26.37 ± 5.94	26.51 ± 5.88	0.851
Fin-foll-up	14.69 ± 4.13	14.61 ± 4.08	0.862

## Discussion

In recent years, TLIF for the treatment of lumbar degenerative diseases has become a widely used surgery. The most important factors are the thorough decompression of nerve and/or cauda equina and the bony fusion of the anatomic anterior column [29]. However, in reconstruction and bony fusion, it is important to avoid complications, such as the failure of internal fixation and cage migration, which is challenging work for orthopedic surgeons [30].

Currently, PEEK cages have been widely used. Lee et al. [18] evaluated the fusion rate of a morselized local bone graft in PEEK cages. They obtained an 86.7 % fusion rate at 6-months and a 90.0 % fusion rate at 12-months follow-up. They believed that 1-year post-operation was a better time point for observing bony fusion. However, the K-ODI, SF-36 and VAS values were similar after surgery. Whether there was a relationship between fusion rate and clinical outcomes remains unknown [18]. Schomacher et al. [31] reported the application of TMCs and PEEK cages for the treatment of pyogenic spondylodiscitis. The solid bony fusion rate was 90.5 % in the PEEK group and 100 % in the TMCs group, but the difference was not significant. Nemoto et al. [8] compared TMCs and PPEK cages in their study and found bony fusion rates of 96 and 64 % at 12-months and 100 and 76 % at 2-years after surgery, respectively. They concluded that there was no demonstrable superiority of PEEK cages over TMCs in regards to bony fusion. Additionally, they found unfavorable vertebral osteolysis in PEEK cages, which may lead to nonunion, and suggested that the improvement in the biocompatibility of PEEK cages was necessary to increase fusion rates [8].

TMCs used for spinal reconstruction have been reported with many disadvantages. For example, Jang et al. [14] found that cage subsidence occurred in 93.3 % of patients after anterior cervical corpectomy and reconstruction, although the fusion rate was 100 %. They thought that cage subsidence could make up for the advantages of TMCs, such as restoration and maintenance of IH, enlargement of the stenotic neural foramen and immediate stabilization of operative segments. Whether there was a relationship between cage subsidence and clinical effects remains unclear. Yang et al. [15] compared TMCs with n-HA/PA66 cages for one-level anterior cervical corpectomy and fusion (ACCF) and observed that the fusion rate of the n-HA/PA66 group was higher than TMCs at 1-year follow-up but that the finial fusion rate was similar. Cage subsidence was significantly lower in the n-HA/PA66 group than in TMCs. The VAS and JOA in the TMCs group were worse than in the n-HA/PA66 group. Zhang et al. [16] reported another comparison between TMCs and n-HA/PA66 cages. According to their study, the fusion rate in the n-HA/PA66 group was higher at the one-year follow-up than the TMCs group for both 1-level and 2-level ACCF, and the cage subsidence was significantly higher in the TMCs group for the 1-level ACCF. Additionally, the difference was significant for the 2-level ACCF between the TMCs and n-HA/PA66 groups.

The n-HA/PA66 cages are made by the Institution of Materials Science and Technology, Sichuan University, and our department. The application was conducted in reconstruction of spine, especially cervical spine. Little articles were used to compare the efficacy of n-HA/PA66 cages with PEEK cages when treating degenerative lumbar diseases. In the present study, we found that the preoperative IH and SA were similar in both the n-HA/PA66 and PEEK groups. Additionally, there were no significant differences in IH and SA at 1-week, 3-, 6-month, 1-year and the final follow-up between the two groups. The correction and loss of IH for the two groups did not differ; neither did the correction of SA. However, the loss of SA was different. In our series, the mean correction of SA in the n-HA/PA66 group was 0.55 ± 5.89°, while the mean correction of SA was greater in the PEEK group. There may be a tendency toward “the more correction, the more loss”, as reported by Rousseau et al. [3]. We thought that loss in lordosis might be related to increased postoperative lordosis and a tendency to recover the initial spinal sagittal balance. However, the fusion and subsidence rates were not affected in our study. Some investigators hold that the loss of IH and SA should be considered a normal and expected result, as slight loss of IH and SA did not affect clinical outcomes [32, 33]. However, others believed that loss of IH and SA were related to the stability and sagittal sequence of the spine and decompression of nerve root and/or cauda equina, especially excessive subsidence [5, 6]. In our report, the VAS and ODI scores increased after operation and did not show a difference between the two groups at any of the time points examined. No failure of internal instrument or pseudarthrosis or obvious vertebral osteolysis was observed during follow-up.

Cage subsidences are influenced by the lower fused segment, cage position, number of fused segments, cage size, amount of morselized bone, end-plate manipulation and the material characteristics of the cage [3, 5–7]. Regarding the cage material characteristics, both n-HA/PA66 and PEEK cages have a low Young’s modulus, similar to natural bone, resulting in lower stress-shielding compared with TMCs [15–17]. In our study, the n-HA/PA66 cage is made from a composite of nano-hydroxyapatite and polyamide66. Hydroxyapatite, a component of natural bone, is nanocrystallized and then well-distributed into polyamide. The composite possesses both the mechanical strength of Hydroxyapatite and the elastic properties of polyamide66. Studies have demonstrated the biocompatibility, safety, osteoconduction and biomechanical stability of n-HA/PA66 fairly well [19–24]. Additionally, the cage shape is characterized by a wide rim with several shallow recesses to prevent cage migration and subsidence via increasing the friction between the cage and end-plate and dispersing pressure on the cage surface. Animal experiments demonstrated that when implanted, the cage can release Ca^2+^ and ${\text{PO}}{_{4}}^{3-}$ from its surface, which gradually forms a crystal layer on the cage surface that bridges the graft and implant bed to provide a trestle for osteogenesis [34]. In addition, the 2 mm holes in the cage walls and grooves theoretically allows the invasion of vessels, growth factors, osteogenic factors and bone morphogenetic proteins to promote bone healing and bony fusion. In the current study, 92.45 % of patients showed bony fusion at the final follow-up. Only 7.55 % of patients suffered from cage subsidence in the n-HA/PA66 group, which was similar to the PEEK group. Meanwhile, the VAS and ODI scores were obviously improved. Considering the high fusion and low subsidence rates similar to other reports [15, 16, 25, 26], we suggest that n-HA/PA66 cages are comparable to PEEK cages as ideal implants for application in TLIF.

Since the application of n-HA/PA66 cages for lumbar spine treatment in 2010, surgeons are expected to gain experience in the following procedures: (1) measure the preoperative IH and choose a suitable cage size; (2) avoid excessive distraction of the intervertebral space, generally 3–4 mm, or the immediate postoperative IH, generally 12–14 mm; (3) maintain the bone end-plate so that it is not broken, while cleaning up the cartilage end-plate completely; (4) ensure sufficient osseous granula to fill the cage; and (5) use a correction of SA that is not great but should correspond to the previous sagittal balance.

Several limitations remain for the present report. Firstly, this was only a case–control and retrospective analysis for the use of n-HA/PA66 and PEEK cages in TLIF. A prospective study is necessary to further confirm the differences observed. Secondly, we selected patients with different diagnoses and segments, which might have some influence on the results. Thus, a layering study should be conducted.

## Conclusions

This case–control and retrospective study demonstrated that the use of both n-HA/PA66 and PEEK cages can promote effective clinical and radiographic outcomes in the treatment of lumbar degenerative diseases with an average 2-year follow-up. The high fusion and low subsidence rates demonstrated that the n-HA/PA66 cage is an alternative ideal substitute material comparable to PEEK cages for lumbar reconstruction after TLIF.
